# Sickle cell hemoglobinopathies in district Bhopal

**DOI:** 10.4103/0971-6866.69373

**Published:** 2010

**Authors:** C. B. S. Dangi, M. Sajid, G. K. Sawke, J. Ambhore

**Affiliations:** Human Genetic Lab, Centre for Scientific Research and Development, People’s Group, Bhanpur, Bhopal, Madhya Pradesh, India; 1 Department of Pathology, People’s College of Medical Sciences, People’s Group, Bhanpur, Bhopal, Madhya Pradesh, India; 2 Department of Pediatrics, People’s General Hospital, Berasia Road Bhopal, Bhopal, Madhya Pradesh, India

Sir,

A hospital based mass screening program conducted in central India to evaluate incidence of Sickle Cell hemoglobinopathies among clinically anemic cases. Primary screening with Naked Eye Single Tube Red Cell Osmotic Fragility Test (NESTROFT) with low values of Red blood cell (RBC) indices like Mean Corpuscular volume (MCV) and mean corpuscular hemoglobin (MCH) were done. Positive results of primary screening were confirmed by secondary screening with the help of cation exchange High Performance Liquid Chromatography (HPLC) (D-10, Bio-Rad), which produces the clear picture of kind of hemoglobinopathies. Initial results were highly encouraging indicating high prevalence rate (7%), in district Bhopal having mixed population of diverse ethnic groups. Among the various form of hemoglobinopathies incidences of S β+/ β0 were highest (45.71%). NESTROFT and HPLC analysis for hemoglobinopathy screening are proved to be highly efficient in present study.

In 1910, Sir James Harric reported peculiar elongated sickle shaped red cells in a severely anemic black student.[1] With passage of time and advancement in technology 698 genetically different hemoglobin variants are found scattered all over the world causing various types of hemoglobinopathies. Sickle Cell anemia is an autosomal recessive inheritant disease. Its clinical severity varies from the milder sickle cell trait (heterozygous) to severe sickle cell anemia (homozygous). Variation in hemoglobin occurs due to substitution of glutamic acid by valine at position six of the b chain of hemoglobin. The clinical manifestation of sickle cell disease (SCD) arises as sickle haemoglobin (HbS or α2βs 2) tends to polymerize at reduced oxygen tension resulting in deformation of red cell into the characteristic rigid sickle shape. Such inflexible red cells cannot pass through microcirculation efficiently, resulting in destruction of the red cell and intermittent vaso-occlusion. It causes anemia, tissue damage and pain.[2] Clinical variations such as death in earlier childhood to a normal life span with few complications. Sickle cell anemia also happens in combination with HbC, HbE or α /β – thalassaemia. Haemoglobinopathies are wide spread all over the world due to migration of peoples from place to place.[3]

Total 500 samples of clinically suspected anemic patients (with hemoglobin ≤ 10 gm/dl.) and age ranging between 3-55 years were randomly selected from the different area of Bhopal district. Screening was done at Department of pathology, People’s College of Medical Sciences and Research Centre (PCMS and RC) and People’s General Hospital, Bhopal. The samples were collected in EDTA vials and immediately subjected to complete blood picture and NESTROFT for primary screening. Only the NESTROFT positive cases with low MCV and MCH were further screened by Electrophoresis and HPLC for qualitative and quantitative estimation of various hemoglobin variants. The hematological parameters such as estimation of hemoglobin, MCV, MCH, Mean Corpuscular Hemoglobin Concentration (MCHC), Packed cell volume(PCV), red blood cell distribution width RDW-CV and Total Red Blood Cell Count were measured using fully automated blood cell counter (PCE – 210, Erma INC, Japan). NESTROFT was performed with in 4 hours after the sample was collected according to standard prescribed method.

Hemoglobin electrophoresis and HPLC were conducted for NESTROFT positive cases with low MCV, MCH and hemoglobin Hb to rule out sickle hemoglobinopathies. It was determined by using cation exchange HPLC. It is a rapid and easy method for qualitative and quantitative determination of various hemoglobin variants.[4]

Out of 500 samples, 35 NESTROFT positive cases with low MCV, MCH were subjected to electrophoresis and HPLC to confirm different types of hemoglobinopathies. Majority of the cases were noted carrying Sβ+ / Sβ0 (45.71%) trait and HbAS (20%). Table 1 represents the hematological and molecular values studied in 35 samples. General clinical symptoms noted in the patients with various forms of hemoglobinopathies were pallor, weakness, fatigue, abdominal pain, joint pain, fever, jaundice, poor appetite and chest pain.

**Table 1:** Laboratory Values of some common Haemoglobinopathies

Laboratory test	AS	SS	AS/HPHF	AS/α	AS/β+	AS/β+
NESTROFT	+ve	+ve	+ve	+ve	+ve	+ve
Hb (g/dl)	8.9	9.1	8.2	9.4	8.1	7.8
HCT (%)	29.88	26.2	27.9	29.1	23.45	31
MCV (fl)	73.4	66.56	70.83	68.6	68.64	73.5
RBC Morphology						
Sickle cells	+	++	++	-	-	+/-
Target cells	+/-	++	-	+/-	+/-	+/-
Microcytosis	++	++	+/-	+	+	+++
Nucleated	+/-	++	+/-	+/-	++	+++
RBC						
Hb Electrophoresis						
HbS	45.32	87.46	70.45	23.4	57.17	49
HbF	1.25	10.53	24.63	0.6	15.52	16.9
HbA	49.44	17.1	1.56	69.2	13.01	31.25
HbA2	3.32	1.26	2.4	2.73	4.19	2.6
Clinical severity	No Symptoms	Moderate/severe	Moderate/severe	Mild/Moderate	Mild/Moderate	Moderate/Severe

Normal Ranges = HbS – 0%, HbF - <1.5%, HbA – 83.4 – 90.79% and HbA2 - <3.5%

Bhopal is situated in the central India having assorted population where Muslims, Sindhies and tribal population are the major carrier of sickle cell gene. In present study, so far 500 anemic patients were studied out of which 35 were suffering from some kind of sickle disorder making roughly 7% of total positive cases. This coincides with the earlier study conducted, indicating high prevalence rate.[5] Our study concludes that most of the cases belong to sickle anemia with b thalassaemia (45.71%) closely following the sickle cell trait (20%). As supported in earlier study this could be due the presence of mixed population.[5] Male / female ratio was almost double which can be accounted for the prevalent socio – cultural condition in our country where more males seek medical attention than females as noted earlier too.[6]

Microcytosis, hypochromia and nucleated RBC’s were detected in various hemoglobinopathies but in present study microcytosis seen maximum in S/β0, where as hyperchromia and nucleated RBC’s maximum in SS, S/β+ and S/β0. Electrophoresis of these cases revealed presence of the average HbS values 87.46% in SS followed by 70.45% in S/HPFH, 57.17% in S/β+, 49% in S/β0 and 23.4% in S/α. Similarly average HbF values were highest in S/HPFH (24.63%) and lowest in S/α (0.6%). Average value of HbA was highest in S/α (69.2%) and lowest in S/HPFH (1.56%). The value of HbA2 was highest in S/β+ (4.19%) followed by AS (3.32%), S/α (2.73%), S/β0 (2.6%) and SS (1.26%) respectively. The clinical symptoms were moderate to severe in SS, S/HPFH and S/β0 followed by mild to moderate in S/α and S/β+. The common clinical features are pallor, weakness, fatigue, abdominal pain, joint pains jaundice, poor appetite and chest pain.

From the present study it is concluded that NESTROFT is an efficient methods for primary screening of hemoglobinopathies at mass scale, along with estimation of various hematological analysis. Further screening with HPLC verifies the result and help in precise diagnosis. The ratio of affected subjects with sickle cell anemia trait is 7% among screened population. The more symptoms were observed in SS followed by S/HPFH and S/β0. The symptoms were subjective varying with age, sex, personal hygiene, socio-economic condition and other associated diseases.
